# Prevalence of SARS-CoV-2 among high-risk populations in Lomé (Togo) in 2020

**DOI:** 10.1371/journal.pone.0242124

**Published:** 2020-11-09

**Authors:** Wemboo Afiwa Halatoko, Yao Rodion Konu, Fifonsi Adjidossi Gbeasor-Komlanvi, Arnold Junior Sadio, Martin Kouame Tchankoni, Koffi Segbeaya Komlanvi, Mounerou Salou, Ameyo Monique Dorkenoo, Issaka Maman, Amétépé Agbobli, Majesté Ihou Wateba, Komi Séraphin Adjoh, Edem Goeh-Akue, Yem-bla Kao, Innocent Kpeto, Paul Pana, Rebecca Kinde-Sossou, Agbeko Tamekloe, Josée Nayo-Apétsianyi, Simon-Pierre Hamadi Assane, Mireille Prine-David, Sossinou Marcel Awoussi, Mohaman Djibril, Moustafa Mijiyawa, Anoumou Claver Dagnra, Didier Koumavi Ekouevi

**Affiliations:** 1 Institut National d’Hygiène (INH), Lomé, Togo; 2 Département de Santé Publique, Université de Lomé, Lomé, Togo; 3 Centre Africain de Recherche en Epidémiologie et en Santé Publique (CARESP), Lomé, Togo; 4 Laboratoire de Biologie Moléculaire et d’Immunologie (BIOLIM), Université de Lomé, Lomé Togo; 5 Faculté des Sciences de la Santé, Université de Lomé, Lomé, Togo; 6 Conseil Scientifique pour la Riposte contre la pandémie de Covid-19, Lomé, Togo; 7 Ministère de la Santé et de l’Hygiène Publique, Lomé, Togo; 8 Coordination Nationale de Gestion de la Riposte contre la Covid-19, Lomé, Togo; 9 Centre Inserm, 1219, Institut de Santé Publique et de Développement et Université de Bordeaux, Bordeaux, France; University of Zambia, ZAMBIA

## Abstract

**Background:**

In December 2019, the COVID-19 outbreak began in China and quickly spread throughout the world and was reclassified as a pandemic in March 2020. The first case of COVID-19 was declared in Togo on March 5. Two months later, few data were available to describe the circulation of the new coronavirus in the country.

**Objective:**

This survey aimed to estimate the prevalence of SARS-CoV-2 in high-risk populations in Lomé.

**Materials and methods:**

From April 23, 2020, to May 8, 2020, we recruited a sample of participants from five sectors: health care, air transport, police, road transport and informal. We collected oropharyngeal swabs for direct detection through real-time reverse transcription polymerase chain reaction (rRT-PCR) and blood for antibody detection by serological tests. The overall prevalence (current and past) of infection was defined by positivity for both tests.

**Results:**

A total of 955 participants with a median age of 36 (IQR 32–43) were included, and 71.6% (n = 684) were men. Approximately 22.1% (n = 212) were from the air transport sector, 20.5% (n = 196) were from the police sector, and 38.7% (n = 370) were from the health sector. Seven participants (0.7%, 95% CI: 0.3–1.6%) had a positive rRT-PCR test result at the time of recruitment, and nine (0.9%, 95% CI: 0.4–1.8%) were seropositive for IgM or IgG against SARS-CoV-2. We found an overall prevalence of 1.6% (n = 15), 95% CI: 0.9–2.6%.

**Conclusion:**

The prevalence of SARS-CoV-2 infection among high-risk populations in Lomé was relatively low and could be explained by the various measures taken by the Togolese government. Therefore, we recommend targeted screening.

## Introduction

In December 2019, an outbreak of pneumonia (COVID-19) due to a new coronavirus first named 2019-nCoV, now officially SARS-CoV-2, occurred in China [1]. In less than five months, this outbreak had spread rapidly to every continent (except Antarctica) with more than 3.7 million people infected and more than 257,000 deaths recorded as of May 8, 2020, in 214 countries and territories [2]. In Africa, 32,953 (0.9%) cases of COVID-19 have been reported as of May 8^th^ 2020 [3].

Since the beginning of the outbreak, health systems in developed countries have faced many challenges in fighting COVID-19. Numerous assumptions have been made about the true magnitude and evolution of the epidemic around the world. It has been commonly assumed that officially reported data are underestimated [4, 5], especially in Africa. The insufficient diagnostic capacity of countries and the high proportion of asymptomatic cases may explain such an underestimation [6]. Thus, the World Health Organization (WHO) has recommended a mass screening strategy for all countries burdened by the epidemic with the hypothesis that [7] more tests performed would result in an easier tracking of the spread of the virus and thus a decrease in transmission [8]. However, there is insufficient testing capacity in many countries due to a high global demand for antibody test kits [8] and GeneXpert which has recently been validated by the US Food and Drug Administration [9]. To date, real-time reverse transcription‐polymerase chain reaction (rRT-PCR) remains the gold standard test for the diagnosis of COVID-19. Antibodies are the best biomarkers to estimate the number of people previously infected and could help estimate the prevalence and inform testing strategies in populations at higher risk of COVID-19.

In Togo, the first case of COVID-19 was reported on March 5, 2020, and as of April 26, 2020, 98 cases were confirmed, including 6 deaths [10]. Only suspected cases, contacts, and travelers were screened for SARS-CoV-2. The value of population mass screening was debated considering the country's relatively limited diagnostic capabilities. Few studies so far have been conducted to estimate the prevalence of SARS-CoV-2 based on rRT-PCR tests or antibody tests including studies in Iceland [11], Santa-Clara County in the USA [12] and Switzerland [13]. To our knowledge, there are no data available on the prevalence of SARS-CoV-2 in sub-Saharan Africa. Based on the low incidence of SARS-CoV-2 infection observed in the general population, the Swiss National Covid-19 Science Task Force recommends focusing research at the population level on subpopulations at higher risk of infection [14]. Therefore, we conducted a pilot survey in high risk populations to estimate the prevalence of SARS-CoV-2 using the rRT-PCR test to refine screening strategies in the fight against the pandemic in Togo.

## Materials and methods

### Study site

A cross-sectional study was conducted by a multidisciplinary team (demographers, epidemiologists, biologists, biostatisticians) among high-risk populations in Lomé (capital city of Togo) from April 23^rd^ to May 8^th^, 2020. Togo is a country of West Africa that covers an area of 56,800 km^2^ with an average density of 145 inhabitants per square kilometer [15, 16]. The population was 7.89 million in 2018, of which 50.2% are women and 60% are under 25 years of age [16]. Lomé is the capital city with a population of approximately 2.1 million in 2017 [17]. Economically, the gross domestic product per Togolese amounted to 682 US dollars in 2019, making Togo the 11th poorest country in the world [18].

### Study design and sampling

Participants were recruited from five professional sectors: healthcare (doctors, nurses, pharmacy auxiliaries, hospital administrators), air transport, police, road transport (taxi drivers) and informal (market sellers and craftsmen). These groups were targeted because they are at high risk of contamination during epidemics due to their high probability of being in close contact with travelers or with COVID-19patients [19, 20]. Participants were eligible to participate in the study if the following four criteria were met: (i) ≥ 18 years of age; (ii) working in one of the five sectors; (iii) having been regularly present at the workstation for the past 30 days; and (iv) living in Lomé for the past 3 months.

Several sampling methods were used for participant selection based on the expected total size of the target population and the availability of a sampling frame. First, exhaustive recruitment was performed among the police (road safety officers) and people in air transport (*International Airport Gnassingbe Eyadema*, *Lomé*, *Togo*). Second, participants from the informal sector were recruited based on an open invitation. All market sellers and administrative staff of one of the selected market in Lomé where invited for SARS-CoV-2 screening. Third, random sampling (two or three stages) was performed for the recruitment of taxi drivers (road transport) and health care workers. For example, for the selection of taxi drivers we performed a two-stage sampling with the selection of the company, and then the selection of the drivers working in the company.

### Sample size estimation

The sample size was estimated using a single proportion population formula [21] with a 95% confidence level, 1% margin of error, and 2% estimated prevalence of SARS-CoV-2 among high risk populations (as defined above) based on surveillance data of travelers in Togo (Ministry of Health). A 10% unusable biological specimen or nonresponse rate was anticipated and the minimum number of participants was estimated at 837.

### Data collection

We established a test site at the ‘*Faculté des Sciences de la Santé de l’Université de Lomé*’ (Faculty of Medicine, University of Lomé) and invited the target population to join us on site for inclusion. After eligibility screening and written informed consent, sociodemographic characteristics and COVID-19 epidemiological data were collected using a standardized questionnaire. The questionnaire was administered by a trained study team (medical doctors) during a face-to-face interview. Oropharyngeal (OP) and blood samples were collected by trained and well-equipped staff of the ‘Institut National d’Hygiène’ (INH) which is the reference laboratory for SARS-CoV-2 testing in Togo. Oropharyngeal swabs were collected using Eswab type swabs (304295 VIRUS FLOCK; DELTALAB, S.L., Plaza de la Verneda 1, 08191 Rubi-Barcelona SPAIN) and samples were tested for SARS-CoV-2 using rRT-PCR at the INH. Whole blood specimens were collected in EDTA tubes to test for anti-SARS-CoV-2 serologic markers at the ‘Laboratoire de Biologie Moléculaire et d’Immunologie, Université de Lomé’ (BIOLIM).

### Laboratory procedures

#### Molecular detection of SARS-CoV-2 using th TIB MOL BIOL rt RT-PCR kit

Detection of SARS-CoV-2 in oropharyngeal samples has been performed using molecular biology methods, such as recommended by WHO and US CDC [22, 23].

The TIB MOLBIOL (Olfert Landt, Berlin Germany) LightMix® SarbecoV E-gene Plus EAV Control PCR Kit and the LightMix® Modular COVID-19 RdRP Gene Kit were used for the amplification and qualitative detection of nucleic acid of SARS-CoV-2, respectively [24]. Amplification was carried out after viral RNA extraction using the QIAamp Viral RNA Mini Kit (Qiagen Str, Hilden, Germany). The detection algorithm used was a two-step process involving first a screening assay for sarbecovirus by targeting the E gene to detect both the SARS virus and the COVID-19 virus; and a second confirmation assay for the COVID-19 virus only targeting the RNA dependent RNA polymerase gene (RdRp), a gene specific for SARS-CoV-2.

Internal quality control was ensured through the use of three controls included in the kit supplied by the company TIB MOL BIOL (Eresburgstr. 22–23 | D-12103 Berlin, Germany). Controls included a control during extraction (EAV extraction control, ref. 40-0776-96, TIB MOL BIOL, Germany) to detect possible inhibition of PCR, a positive control for each gene to be detected (positive control of the E gene, ref. 40-0776-96, TIB MOL BIOL, Germany and positive control of the RdRp gene; ref. 53-0777-96, TIB MOL BIOL, Germany) to ensure optimal performance of PCR reaction and a negative control (no control model, NTC) to verify the absence of contamination of the reagents. The validity of the test was only accepted if the cycle threshold (Ct) value of the E positive gene control was <30, the positive control of RdRp gene was <30, and control of the extraction of the EAV was <33 and if the NTC did not generate an amplification curve.

A sample was considered positive if the Ct values of E <36 and RdRp <40 with the presence or absence of the EAV extraction control. If the RdRp gene was not observed or the Ct> 40, the sample was considered probable for COVID-19. A sample was considered negative (below the threshold) if no amplification curve was observed for the E and RdRp genes and if the Ct value for the EAV extraction control was <33. If, on the other hand, the EAV had no curve, the test was rerun for the corresponding sample.

The INH molecular biology laboratory participated in May 2020 in external quality control under the supervision of the WHO within the framework of its pilot program external quality assessment program (EQAP) for the detection of SARS-CoV-2 virus by RT-PCR.

#### Detection of SARS-CoV-2 antibodies

Detection of serological markers (immunogobulins G and M) of SARS-CoV-2 infection was carried out at BIOLIM using the Lungene® Rapid Test (Hangzhou Clongene Biotech Co, Ltd). Serum was used for testing. Ten microliters of serum was added dropwise to the well on the plastic plate, and after adding two drops (50 to 70 microliters) of buffer, the presence of specific IgM and/or IgG isotype antibodies in the body was read based on the strips in the test field. The test result was validated when the control band (C) appeared: for a visible control band, a band of any strength appeared in the IgM field anti-SARS-CoV-2 anti-IgM, and the presence of anti-SARS-CoV-2 anti-IgG was detected in the band of any strength in the IgG field. This procedure was followed according to the manufacturer’s instructions and as described in the literature [25]. The sensitivity and specificity were 72.85% and 85.02%, respectively [25].

This test was also validated by the Laboratory Department of the Ministry of Health in Togo. The sensitivity of the assay using samples from participants previously diagnosed with COVID-19 on the day of hospitalization was 77.1% for IgM or IgG, and the specificity was 95.4%. Moreover, the sensitivity measured 7 days after hospitalization was 93.3% for IgG signing the contact with SARS-CoV-2 [26].

### Care and treatment

Biological sample test results were available within 48 hours. All participants who screened positive for SARS-CoV-2 were quarantined in a dedicated hotel or at the national COVID-19 treatment center and those who tested negative were invited to respect all the mitigation measures proposed by the government.

### Statistical analysis

Descriptive statistics were performed and the results are presented with frequency tabulations and percentages for categorical variables. Quantitative variables are presented as medians with their interquartile range (IQR). Seroprevalence of antibodies against SARS-CoV-2, prevalence of SARS-CoV-2 infection by rRT-PCR and overall prevalence of past or current infection (positive rRT-PCR or antibody seropositivity) were estimated, along with their 95% confidence interval (95% CI). Comparisons of categorical variables were performed using chi-square or Fisher’s exact tests. Data analyses were performed using R^©^ version 3.4.3 software and the level of significance was set at 5%.

### Ethical considerations

Ethical approval was obtained from the *‘Comité de Bioéthique de Recherche en Santé’* (Bioethics Committee for Health Research) from the Togo Ministry of Health (No. 004/2020/CBRS). Potential participants were informed about the study purpose and procedures, potential risks and protections. Those willing to participate were invited to sign a consent prior to participation.

## Results

### Sociodemographic characteristics

A total of 955 people with a median age of 36 (IQR 32–43) were included in the study and 71.6% (n = 684) were men. Approximately 22.1% (n = 212) were in the field of air transport, 20.5% (n = 196) in the police, 5.8% (n = 55) in the informal sector, 38.7% (n = 370) in the health sector and 12.8% (n = 122) in the road transport sector. None of the participants had been previously diagnosed as COVID-19 positive or hospitalized in the last 30 days before enrollment. The majority of participants, (n = 936, 98.0%) were Togolese, approximately two-thirds (n = 636, 66.6%) were part of a couple, and half (n = 487, 51.0%) of them had a university level degree. The sociodemographic characteristics according to the sector of activity are summarized in Table 1.

**Table 1:** Sociodemographic characteristics according to sector of activity, Lomé, Togo.

	Air transport	Police	Informal sector	Health sector	Road transport	Total	p-value
	n = 212	n = 196	n = 55	n = 370	n = 122	N = 955	
**Age (years)**							<0.001*
<36	90 (42.5)^£^	121 (61.7)	17 (30.9)	158 (42.7)	48 (39.3)	434 (45.4)	
≥36	122 (57.5)	75 (38.3)	38 (69.1)	212 (57.3)	74 (60.7)	521 (54.6)	
**Sex**							<0.001^$^
Men	179 (84.4)	168 (85.7)	34 (61.8)	181 (48.9)	122 (100.0)	684 (71.6)	
Women	33 (15.6)	28 (14.3)	21 (38.2)	189 (51.1)	0 (0.0)	271 (28.4)	
**Nationality**							0.018^$^
Togolese	205 (96.7)	196 (100.0)	52 (94.5)	364 (98.4)	119 (97.5)	936 (98.0)	
Others	7 (3.3)	0 (0.0)	3 (5.5)	6 (1.6)	3 (2.5)	19 (2.0)	
**In couple**							<0.001*
No	49 (23.1)	49 (25.0)	10 (18.2)	169 (45.7)	42 (34.4)	319 (33.4)	
Yes	163 (76.9)	147 (75.0)	45 (81.8)	201 (54.3)	80 (65.6)	636 (66.6)	
**Education level**							<0.001^$^
None	3 (1.4)	0 (0.0)	5 (9.1)	5 (1.4)	11 (9.0)	24 (2.5)	
Primary	2 (0.9)	1 (0.5)	4 (7.3)	11 (3.0)	26 (21.3)	44 (4.6)	
Secondary	105 (49.5)	143 (73.0)	15 (27.3)	69 (18.6)	68 (55.7)	400 (41.9)	
University	102 (48.1)	52 (26.5)	31 (56.4)	285 (77.0)	17 (13.9)	487 (51.0)	

£: percentages

*: Pearson Chi-square test

$: Fisher test

### Prevalence of current infection as determined by a positive rRT-PCR

Seven participants (0.7%, 95% CI: 0.3–1.6%) had a positive rRT-PCR test for SARS-CoV-2 (Table 2) at the time of recruitment and the prevalence varied from 0% for the participants from road transport to 1.8% for those in the informal sector of activities. The prevalence was 0.7%, 95% CI [0.3–1.8] in men and 0.7%, 95% CI [0.1–2.8] in women (p = 0.683, Fisher’s exact test).

**Table 2:** Prevalence of SARS-Cov-2 according to sector of activity in Lomé, Togo.

	Air Transport (n = 212)		Police (n = 196)		Informal Sector (n = 55)		Health (n = 370)		Road transport (n = 122)		Total (N = 955)	
	n (%)	95%CI	n (%)	95%CI	n (%)	95%CI	n (%)	95%CI	n (%)	95%CI	N (%)	95%CI
**PCR+**	3 (1.4)	[0.3–4.1]	1 (0.5)	[0.0–2.8]	1 (1.8)	[0.04–9.7]	2 (0.5)	[0.06–1.9]	0 (0.0)	[0.0–2.9]	7 (0.7)	[0.3–1.6]
**IgM+**	1 (0.5)	[0.01–2.6]	1 (0.5)	[0.0–2.8]	0 (0.0)	[0.0–6.5]	0 (0.0)	[0.0–1.0]	0 (0.0)	[0.0–2.9]	2 (0.2)	[0.03–0.8]
**IgG+**	2 (0.9)	[0.1–3.4]	0 (0.0)	[0.0–1.9]	0 (0.0)	[0.0–6.5]	5 (1.4)	[0.4–3.1]	1 (0.8)	[0.0–4.5]	8 (0.8)	[0.4–1.7]
**PCR+ or IgG+ or IgM+**	4 (1.9)	[0.5–4.8]	2 (1.0)	[0.1–3.6]	1 (1.8)	[0.04–9.7]	7 (1.9)	[0.8–3.9]	1 (0.8)	[0.0–4.5]	15 (1.6)	[0.9–2.6]

+: Positive 95%CI: 95% Confidence interval

### Seroprevalence of antibodies against SARS-CoV-2

**Fig 1:**
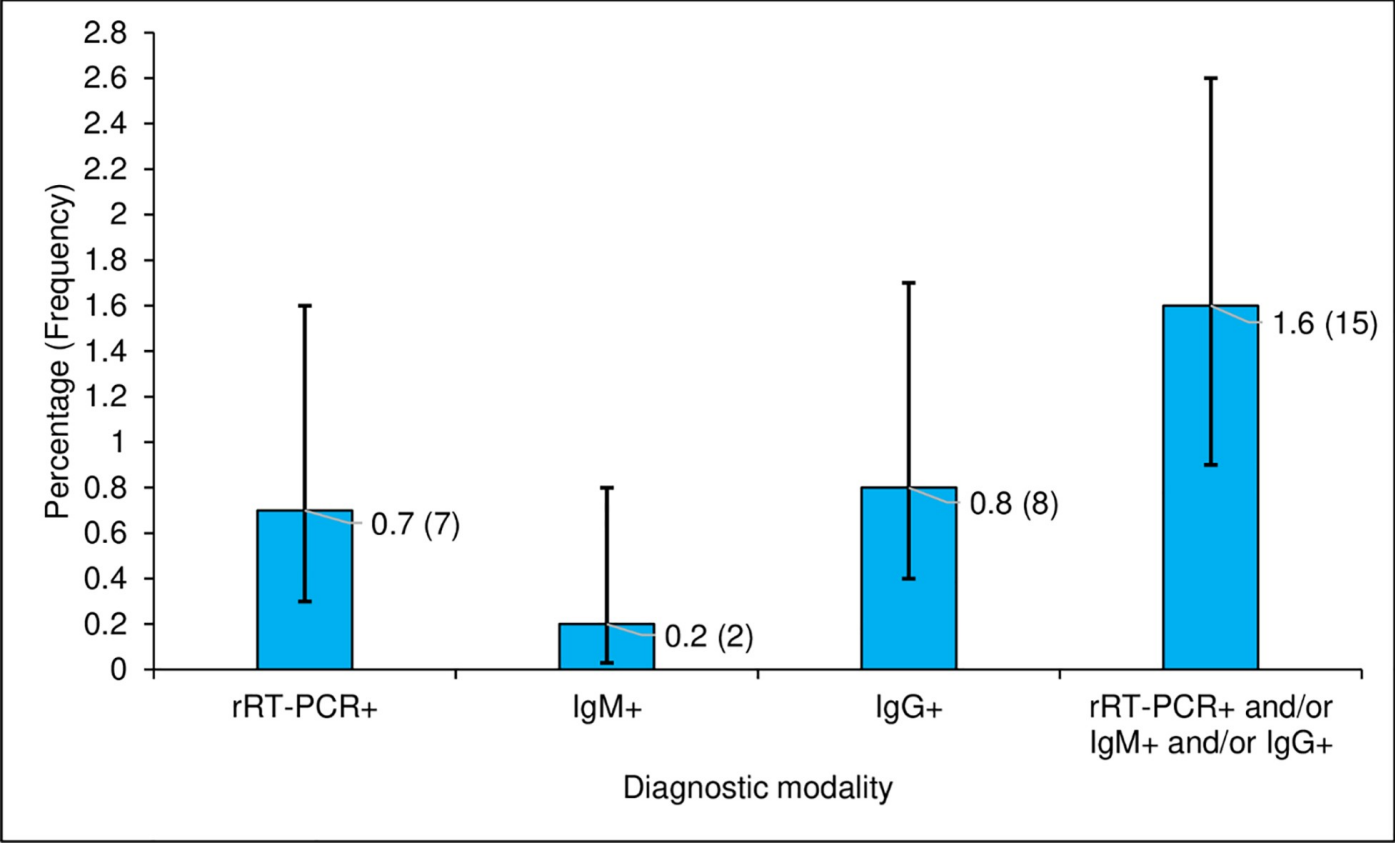
Prevalence of SARS-CoV-2 based on rRT-PCR test and serological test in high risk population, Lomé (Togo).

Nine participants (0.9%, 95% CI: 0.4–1.8%) were seropositive for IgM or IgG against SARS-CoV-2 (Table 2 and Fig 1) and one of them was seropositive for both IgM and IgG. Table 2 summarizes the seroprevalence of IgG or IgM according to the different sectors. Thus, a total of 15 participants (1.6%, 95% CI: 0.9–2.6%) were positive for rRT-PCR or seropositive for IgM or IgG against SARS-CoV-2. This prevalence ranged between 0.8% in the road transport sector and 1.9% in the health sector (Table 2).

### Clinical manifestations, care and treatment

Six out of seven rRT-PCR positive participants were asymptomatic. The symptomatic participant had presented fever, headaches, and myalgia during the two weeks prior to enrollment. All rRT-PCR positive participants were hospitalized at the national reference center for COVID-19 and treated with hydroxychloroquine and azithromycin as recommended by the national guidelines [27]. A description of the seven rRT-PCR positive participants is presented in Table 3.

**Table 3:** Description of rRT-PCR and serology positive participants (N = 15).

	Age (years)	Sex	Sector of activity	rRT-PCR	IgG +	IgM +	Covid-19 symptoms
Participant 1	58	Male	Air transport	**+**	No	No	No
Participant 2	52	Male	Air transport	**+**	No	No	No
Participant 3	35	Male	Air transport	**+**	**Yes**	No	No
Participant 4	37	Male	Health	**+**	No	No	No
Participant 5	30	Female	Health	**+**	No	No	Yes*
Participant 6	52	Male	Informal	**+**	No	No	No
Participant 7	32	Female	Police	**+**	No	No	No
Participant 8	46	Female	Health	-	**Yes**	No	Yes*
Participant 9	42	Male	Health	-	**Yes**	No	No
Participant 10	26	Male	Transport routier	-	**Yes**	No	No
Participant 11	37	Female	Health	-	**Yes**	No	No
Participant 12	53	Female	Health	-	**Yes**	No	Yes*
Participant 13	56	Male	Health	-	**Yes**	No	No
Participant 14	64	Male	Air transport	-	**Yes**	**Yes**	No
Participant 15	38	Male	Police	-	No	**Yes**	Yes*

rRT-PCR: Real time polymerase chain reaction, IgG: Immunoglobulin G, IgM: Immunoglobulin M, +: Positive, *Presence of fever, headache and myalgia within the past two weeks before enrolment

## Discussion

This study reported the prevalence of SARS-CoV-2 in sub-Saharan-Africa in a representative sample of high-risk populations. The present study was conducted in the context of the urgent need for data for decision-making and refinement of response strategies. SARS-CoV-2 prevalence was assessed by molecular biology and serologic tests. In Lomé, the capital city of Togo, two months after the first case of COVID-19, the prevalence of SARS-CoV-2 infection among persons at high risk for infection was 1.6% based on the presence of antibodies and viral genomes. Using rRT-PCR alone, only 0.7% of the study population was found to be infected with SARS-CoV-2.

Few studies have focused on high risk populations for COVID-19. In the United States, the screening of an alleged high-risk population of residents and staff members from five homeless shelters was conducted in the cities of Boston, San Francisco, Seattle and Atlanta [28]. This study reported a prevalence of SARS-CoV-2 infection ranging from four to 66% among residents and one to 30% among staff [28]. Healthcare workers (HCWs) are considered particularly high-risk populations. In a study conducted in the United Kingdom (UK), over a 3-week period (April 2020), 1,032 asymptomatic HCWs were screened for SARS-CoV-2 in a large UK teaching hospital. rRT-PCR was used to detect viral RNA from a throat and nose self-swab [29]. Among these asymptomatic HCWs, 3% tested positive for SARS-CoV-2 [29]. In Lomé, the prevalence among HCWs was 0.5% based on virological tests and 1.9% based on both virological and antibody tests, but this included administrative and pharmacy staff.

Most COVID-19 prevalence surveys have been carried out in the general population. In a survey conducted in Iceland, 1,221 (13.3%) of the 9,199 people who were recruited using the symptom-targeted method were positive for SARS-CoV-2 infection [11]. Among those tested by open invitation selection or random selection, SARS-CoV-2 prevalence was 0.8% and 0.6%, respectively [11]. In another survey in Santa Clara County, California, USA, the crude prevalence of SARS-CoV-2 antibodies was 1.5% [12]. After weighing for population demographics of Santa Clara County, the prevalence was 2.8% [12]. In Geneva, Switzerland, another study found that the seroprevalence of SARS-CoV-2 in the general population was low (approximately 5%), despite the high incidence of COVID-19 in Geneva compared with other cantons [13]. All of the studies carried out in the general population based on surveys or mathematical models such as in France (4.4%), reported low prevalence of SARV-CoV-2 despite the magnitude of the infection [30].

Recent evidence highlighted the highest sensitivity of the use of nasopharyngeal samples compared to oropharyngeal samples in rRT-PCR [31]. In a study conducted in China including 353 patients, using both oropharyngeal and nasopharyngeal swabs, SARS-Cov-2 rRt-PCR was positive in 19.0% of nasopharyngeal specimens against 7.6% in oropharyngeal specimens [32]. Another survey reported that the SARS-CoV-2 detection rate was significantly higher for nasopharyngeal swabs [46.7% (56/120)] than oropharyngeal swabs [10.0% (12/120)] (P < 0.001) [31]. This could certainly contribute to underestimating the prevalence reported in our population. This prevalence could be multiplied by two or three according to available data but remained less than 3%. However, at the time of the survey there was no clear recommendation on which swab to choose. Additionally, the unavailability of nasopharyngeal swabs did not allow us to collect both specimens.

The use of serological tests as an effective method for the detection of SARS-CoV-2 has been reported previously in the literature. In a study conducted in Wuhan, China, of the 56 enrolled symptomatic patients, 40 (71%) showed negative nucleic acid tests and 16 (29%) were positive. Among the 40 negative patients, 34 (85%) tested positive for the presence of IgM antibodies. Among the 16 patients who tested positive with nucleic acid tests, one patient showed a negative IgM level. The IgG antibody test was positive in all 56 patients [33]. In the present study, the prevalence of SARS-CoV-2 increased to 1.6% when serology results were included in the analysis. Based on the validation of the test in Togo and elsewhere, the data reported on serological samples seem reliable. Since serology is rapid and inexpensive it could constitute an attractive screening alternative for countries with limited resources in the context of easing of restrictive measures.

This study targeted people considered at high risk for COVID-19 based on professional activity as recently recommended by the Swiss National Covid-19 Science Task Force. The WHO also recommends conducting a survey to estimate the prevalence of SARS-CoV-2 in the community, and for critical population subgroups such as nursing homes or health care facilities [34].

Some of our results were contradictory to the positivity kinetics of rRT-PCR and SARS-CoV-2 antibodies reported in the literature [35]. “Participant 3" (Table 3) was positive for rRT-PCR and IgG but negative for IgM. For this participant, IgM should have been positive. In addition, "Participant 15" was negative for rRT-PCR and IgG but positive for IgM. The latter should have been IgG positive. This could be explained by the intrinsic performance (sensitivity and specificity) of the serological test. In addition, possible cross-reactions with antibodies of other pathogens (plasmodium, other coronaviruses) cannot be excluded. Further studies are needed for a better description of the sensitivity or serological tests.

This study has some limitations. First, the included population was not representative of the general Togolese population, and we actively chose this selection criterion to have a high probability of identifying cases of SARS-CoV-2 infection. Second, we cannot exclude selection bias in our sample due to the recruitment methods. We recruited more participants from the health sector. This could be explained by the diversity of the professions (physicians, pharmacists, administrators) included in this sector. Furthermore, recruitment in the informal sector was difficult due to the lack of a sampling frame and the poor organization of this sector. Future studies should better target these populations and use other recruitment methods, such as quota recruitment. The difference in sector sample size could have affected the overall prevalence which should be interpreted with caution. Based on the low prevalence, we did not study the association between the prevalence of SARS-CoV-2 according to the different sectors. Finally, this study was conducted only in Lomé, the capital city of Togo, where 55% of COVID-19 cases were identified at the time of the survey.

## Conclusion

The prevalence of SARS-CoV-2 infection in the capital city among high-risk populations was relatively low two months after the notification of the first case. The low circulation of the virus in high-risk populations could be explained by the various measures taken by the Togolese government. Based on this result, generalized screening of SARS-CoV-2 would be time-consuming, not cost effective and at a high risk of consuming reagent. Therefore, we recommend a targeted approach for screening. Targeted screening could include health professionals, airport staff; teachers could also be considered in the event of the reopening of schools. Repeated prevalence surveys are needed to refine the strategies to fight against COVID-19 in Togo.

## Supporting information

S1 Dataset (XLSX)

## List of abbreviations

95% CI: 95% confidence interval
IQR: interquartile range
